# Potential values of circulating tumor cell for detection of recurrence in patients of thyroid cancer: a diagnostic meta-analysis

**DOI:** 10.1186/s12885-022-09976-5

**Published:** 2022-09-05

**Authors:** Ming-Xing Liang, Yin-Jiao Fei, Kai Yang, Wen-Juan Tang, Xin-Hui Cao, Jin-Hai Tang

**Affiliations:** 1 Department of General Surgery, the First Affiliated Hospital of Nanjing Medical University, 210029 Nanjing, P.R. China; 2 School of Clinical Medicine, Xuzhou Medical University, 221000 Xuzhou, P.R. China

**Keywords:** Circulating tumor cell, Thyroid cancer, Recurrence, Diagnosis, meta-analysis

## Abstract

**Background:**

Several studies have reported that circulating tumor cells (CTCs) are a promising marker for the diagnosis of thyroid cancer (TC) with recurrence or distant metastasis (DMs). However, some studies emerged with conflicting results. Therefore, we provide a meta-analysis to evaluate the diagnostic performance of CTC for detection of recurrence in patients of TC.

**Methods:**

We searched PubMed, Web of Science, Cochrane library with the keywords “thyroid cancer” and “circulating tumor cells”. Data extraction and risk of bias assessment were performed independently by two reviewers. The summary receiver operating characteristic curve (SROC) and other parameters were adopted to summarize the overall test performance. The sensitivity of CTCs in the detection of recurrent TC was reviewed. All analyses were performed by STATA 12.0 and Meta-disc software.

**Results:**

For CTCs expressing epithelial cell adhesion molecule (EpCAM), seven studies were included in our meta-analysis. Pooled sensitivity, specificity, and diagnostic odds ratio were 0.71 (95% CI: 0.63–0.78), 0.89 (95% CI: 0.84–0.94), and 26.75 (95% CI: 9.11–78.53); 0.78 (95% CI: 0.65–0.89), 0.88 (95% CI: 0.76–0.96), and 40.01 (95% CI: 10.49–152.63) for CTCs expressing thyroid stimulating hormone receptor (TSHR). The area under the SROC for EpCAM and TSHR were both 0.91.

**Conclusion:**

CTC was a reliable marker for the diagnosis of TC patients with recurrence and DMs, and the sensitivity of CTCs expressing TSHR was higher than that of EpCAM. Additional research is warranted in order to establish uniformity in international guidelines, make up the drawbacks of conventional diagnostic methods and to prevent futile surgery.

## Background

Thyroid carcinoma (TC) represents the most common endocrine malignancy and is characterized as one of the most rapidly increasing malignant tumors in recent years, accounts for approximately 2.1% of all cancer cases diagnosed worldwide [1]. The increasing incidence is indicated by the annual percent change (APC) that in the USA women was 7% between 1998 and 2012 [2]. Thyroidectomy and postoperative radioactive iodide (^131^I) therapy is the most fundamental and effective methods of treatment, with a favorable 10-year overall survival rate. However, about 6%~24% of patients remain at risk of tumor recurrence for papillary thyroid cancer. Regional recurrence and distant metastasis (DMs) during the first year after initial thyroidectomy are poor prognostic factor, considered to be life-threatening [3].

Routine surveillance of the disease status mainly includes serum thyroglobulin (Tg), and medical imaging such as ultrasonography, computed tomography (CT), magnetic resonance imaging (MRI), and ^131^I-whole body scintigraphy (^131^I-WBS) [4]. However, Tg testing always needs to be performed with thyroid stimulating hormone suppressive therapy suspended and patients often suffer from severe hypothyroidism and few of them can tolerate it. In addition, Tg determination can be interfered by the presence of anti-Tg antibody (anti-TgAb), while radioactive iodine is essential for scintigraphic imaging analysis and have potential adverse effects [5]. Of note, there is an interval time between surgery and the traditional monitoring methods, which could be critical for catching the early recurrence. Therefore, developing new biomarkers to monitoring disease status is warranted.

Circulating tumor cells (CTCs), namely circulating epithelial cells (CECs), defined as the “break away” cancer cells in the peripheral blood of cancer patients, were first proposed by Ashworth in1860s and further confirmed afterwards [6]. CTCs, shedding from the tumors, circulating in the blood, and reaching different locations, were deemed as the main cause of DMs [7]. As a potential diagnostic biomarker for malignant tumor, peripheral CTCs were characterized by minimal invasion and convenience compared with conventional diagnostic method, such as ^131^I WBS and fine needle aspiration. Assessment of CTCs has been proved to monitor treatment responses and disease status in multiple tumors, such as lung, breast, and colorectal cancers [8–10]. TC is a tumor of epithelial origin and CECs could be also considered as CTCs in pathologically diagnosed TC cases [11]. The prognostic value of CTCs expressing mainly two types of surface or intracellular proteins includes the epithelial cell surface marker epithelial cell adhesion molecule (EpCAM) and thyroid stimulating hormone receptor (TSHR). Therefore, the number of CTCs expressing EpCAM or TSHR could clearly identify patients with recurrence or DMs. Assays for CTCs establish a new and effective approach to reflect the invasiveness of the tumor, and provide clinicians valuable diagnostic information.

In recent years, there have been some reports of CTCs in the diagnosis of TC. However, the system evaluation of CTCs as a diagnostic biomarker for TC is deficient and considerable heterogeneity exists. So, we made this first diagnosis meta-analysis to summarize the evidence of the potential prognostic value of CTCs expressing EpCAM or TSHR in TC.

## Methods

### Search strategy

A literature search for relevant studies was systematically performed by two researchers independently. Any disagreement in study selection was resolved by discussion or arbitration by a third reviewer. We searched PubMed, Web of Science, Cochrane library and Google Scholar database with key words “thyroid cancer”, “thyroid carcinoma”, “thyroid neoplasms”, “thyroid tumors”, “circulating tumor cells”, “CTCs”, “Circulating epithelial cell” and “CECs”. For more comprehensive analysis, the enrolled articles had no restriction of language and time. In order to prevent relevant studies missed, “related articles” function of PubMed was adopted to identify other potentially relevant literatures.

### Methodological quality assessment

Methodological quality of the included studies were assessed by employing Quality Assessment of Diagnostic Accuracy Studies-2 (QUADAS-2) [12]. QUADAS-2 is made up of four components: patient selection, index test, reference standard, and flow and timing. All components were evaluated in terms of bias risk and each component has iconic questions aiming at helping evaluators to judge bias risk accurately. In the evaluating process, if the information obtained from the studies was correspond with the criteria of QUADAS-2, one point was awarded. Otherwise, scoreless was recorded.

### Inclusion and exclusion criteria

In order to ensure the accuracy and reliable of analysis, studies were selected for meta-analysis if the following inclusion criteria were met: (1) investigating the association between the CTCs and recurrence or metastases of TC, regardless of level of reporting; (2) sufficient data was obtained in order to extract the number of true positives (TP), true negatives (TN), false positives (FP), and false negatives (FN); (3) definite pathological diagnosis or medical imaging was available as a reference standard and (4) at least 20 patients were involved in the studies. The exclusion criteria were (1) studies based on overlapping patients; (2) meta-analysis, review, single test, case report, reporting of the expert experience; (3) result is not clear or obvious paradox exists.

### Data extraction

One reviewer (L.M.X) assessed titles and abstracts of studies retrieved by the search strategy for potential eligibility. Another reviewer (F.Y.J) evaluated the accuracy of decision by randomly rescreen sample of 10%. After the elimination of irrelevant articles, two reviewers (C.X.H and Y.K) independently examined full-text studies that met the inclusion criteria. Discrepancies were resolved by the third author (T.W.J). Data extracted from the studies included name of first author, year of publication, country, study design, number of patients, detection markers, methods for CTCs detection and diagnostic criteria and cut-off values. TP, TN, FP, and FN were also retrieved from each enrolled study to calculate the values of pooled sensitivity and specificity. When raw data on diagnostic accuracy was not directly provided in the original articles, study authors were contacted in order to obtain additional data.

### Statistical analysis

For each study included in the meta-analysis, data were extracted to construct 2 × 2 tables displaying TP, TN, FP and FN. Summary estimates, including pooled sensitivity, specificity, and diagnostic odds ratio (DOR), were calculated with corresponding 95% confidence interval (CI). The sensitivity was defined as the proportion of people with a target disease who presented a positive detection result, while the specificity was defined as the proportion of people without the target disease who showed a negative detection result. The DOR enjoyed the advantage of being independent of disease prevalence and was calculated as TP/ FN over FP/TN [13]. If the DOR value of the test is above one, the diagnostic test was deemed to be discriminative. Summary receiver operating characteristic (SROC) curve was also plotted to elucidate the overall performance of the index tests. The closer the curve approaches the top left corner of SROC curve, the higher the overall performance was given [14]. The heterogeneity of sensitivity and specificity across the studies was assessed by chi-squared-based Cochran’s Q statistic test and *I*^2^ inconsistency test. If *I*^2^ > 50% or *P* < 0.1, significant heterogeneity exists and the random effect model was applied, otherwise the fix random model was used. Our meta-analysis was implemented with Stata software, version 12.0 (Stata Corp, College Station, TX, USA) and Meta-Disc software, version 1.4 (Universidad Complutense, Madrid, Spain). A *p* value of < 0.05 was considered statistically significant.

## Results

### Characteristics and quality of the included studies

**Fig. 1:**
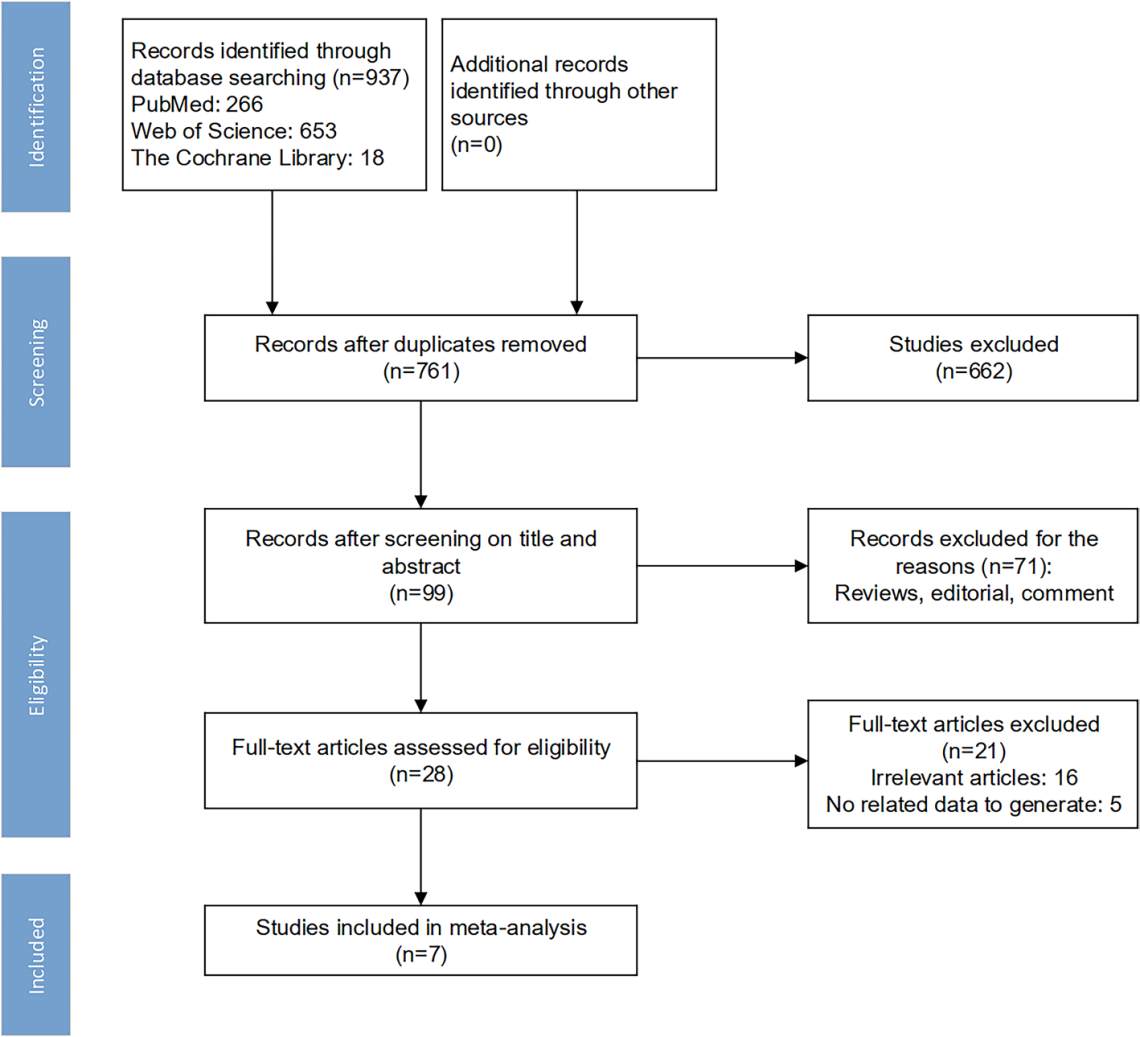
Flow chart of selection process for eligible studies. Total 937 records were searched on the platform of PubMed, Web of Science and The Cochrane Library. After screening title, abstract and full-text in every record, we included 7 eligible studies for further meta-analysis

**Fig. 2:**
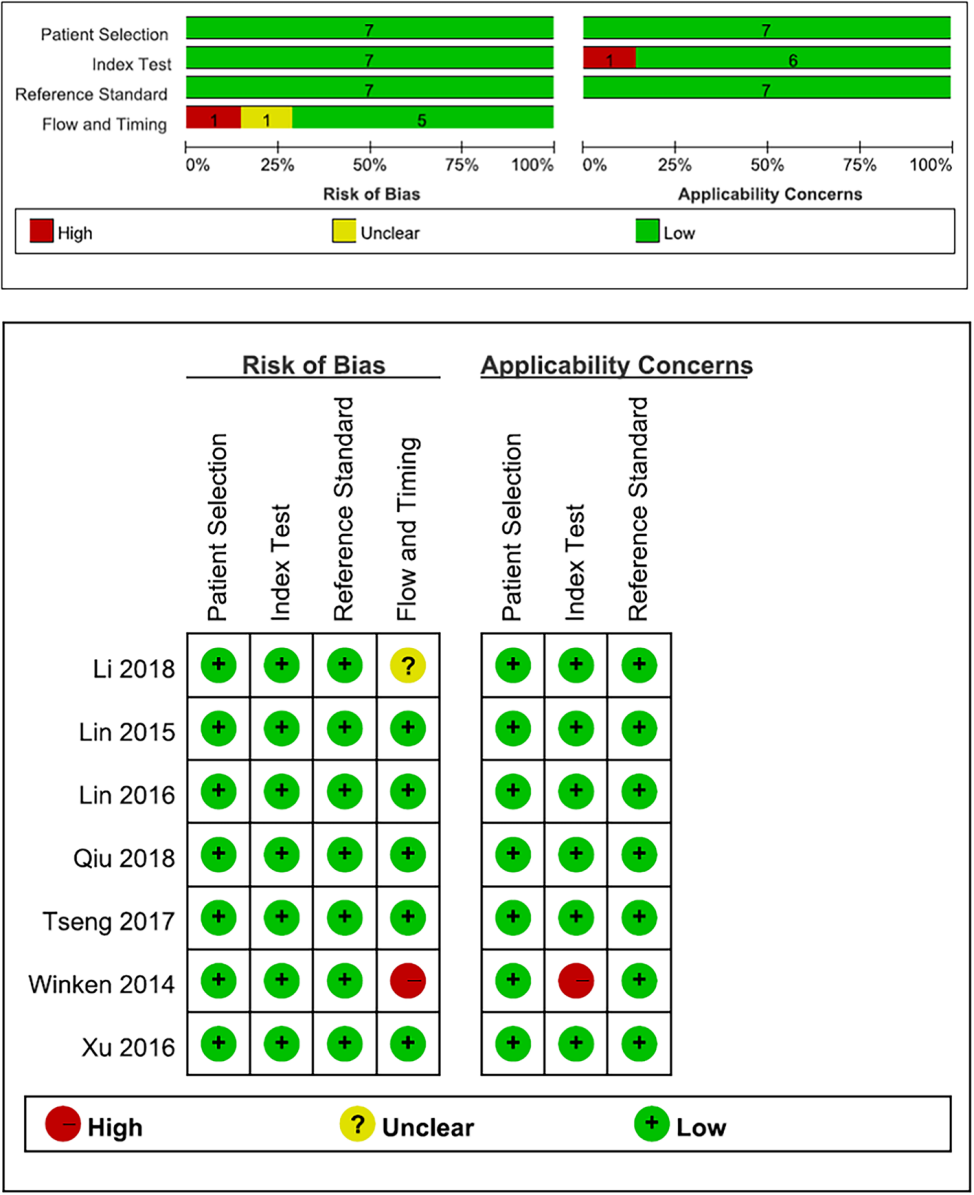
Quality assessment of included studies by using the QUADAS-2 tool. Integrating system of four parts in QUADAS-2 tool, namely patient selection, index test, reference standard, and flow and timing, divided the 7 studies quality assessment into low, unclear and high level. The above graph depicted whole quality level of included 7 studies. And the below pattern stated evlauation process of every study in detail

Figure 1 depicts the flowchart of the literature search, and Table 1 presents the detailed characteristics of the studies. Initially, 937 relevant studies were identified in the systematic literature search. A flowchart of the detailed selection steps was provided in Fig. 1. By checking the titles and abstracts, 662 studies were excluded due to unrelated topics and 99 potential studies were retrieved. An additional 71 studies were then excluded after they were fully reviewed for the following reasons: narrative review/editorial/ comment. Finally, 7 studies were yielded as meeting our inclusion criteria and were eligible for our study (Fig. 1). The 7 included studies were published between 2014 and 2018. All studies detected tumor cells from peripheral blood with the molecular detection method of immunofluorescence or CellSearch. In addition, all of the studies were prospective design. The methodological quality of the studies assessed by the QUADAS-2 tool is depicted in Fig. 2, and the overall methodological quality was better

**Table 1:** Main characters of the studies included in the meta-analysis [15–21]

Authors	Year	District	Study design	participants	Reference standard	Detection method	Biomarkers	TP	FP	TN	FN
Lin et al.	2018	Taiwan	Prospective	48	Cytopathologically	Immunofluorescence	EpCAM、TSHR	22	2	4	20
Xu et al.	2016	USA	Prospective	42	Medical image	CellSearch	EpCAM	13	0	19	10
Tseng et al.	2017	Taiwan	Prospective	77	Medical image	immunofluorescence	EpCAM	27	2	5	43
Li et al.	2018	Taiwan	Prospective	25	Medical image	immunofluorescence	EpCAM、TSHR	6	0	1	18
Qiu et al.	2018	China	Prospective	72	Pathological、medical image	immunofluorescence	EpCAM	19	7	11	35
Winken et al.	2014	Germany	Prospective	14	Tg、Medical image	immunofluorescence	EpCAM	1	4	1	8
Lin et al.	2015	Taiwan	Prospective	29	Biochemical、medical image	immunofluorescence	EpCAM、TSHR	16	2	2	9

*TP* true positive, *FP *false positive, *TN *true negative, *FN *false negative

### Overall analysis

**Fig. 3:**
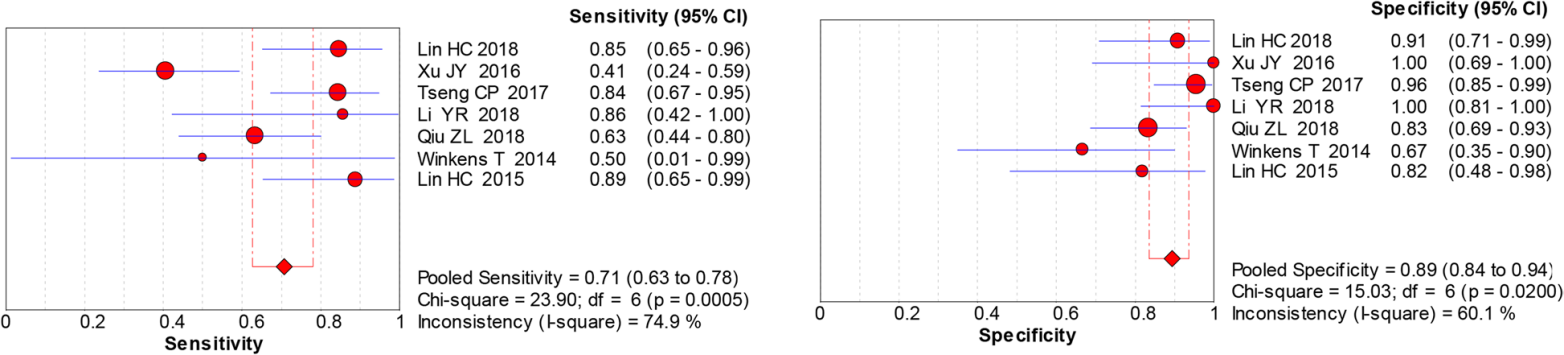
Forest plot of reported sensitivity and specificity of epithelial cell adhesion molecule (EpACM). Left forest plot was for sentivity of EpACM and right forest plot was for specificity of EpACM in studies. Size of red circle mean included sample numbers of every study. And extended blue line represented their 95% CI range. The bottom rhombus was calculated as whole sensitivity or specificity value of included studies. And red hatched line was used to compare 95% CI of whole value to respecitive value in every single study

**Fig. 4:**
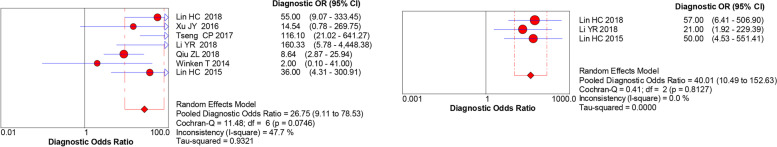
Forest plot showing the both pooled diagnostic odds ratio. Left forest plot was for diagnositic odds ratio of EpACM and right forest plot was for diagnositic odds ratio of TSHR in corresponding studies. The blue arrows on the right indicated the above values of 95% CI were exceeded the maximum of the abscissa

**Fig. 5:**
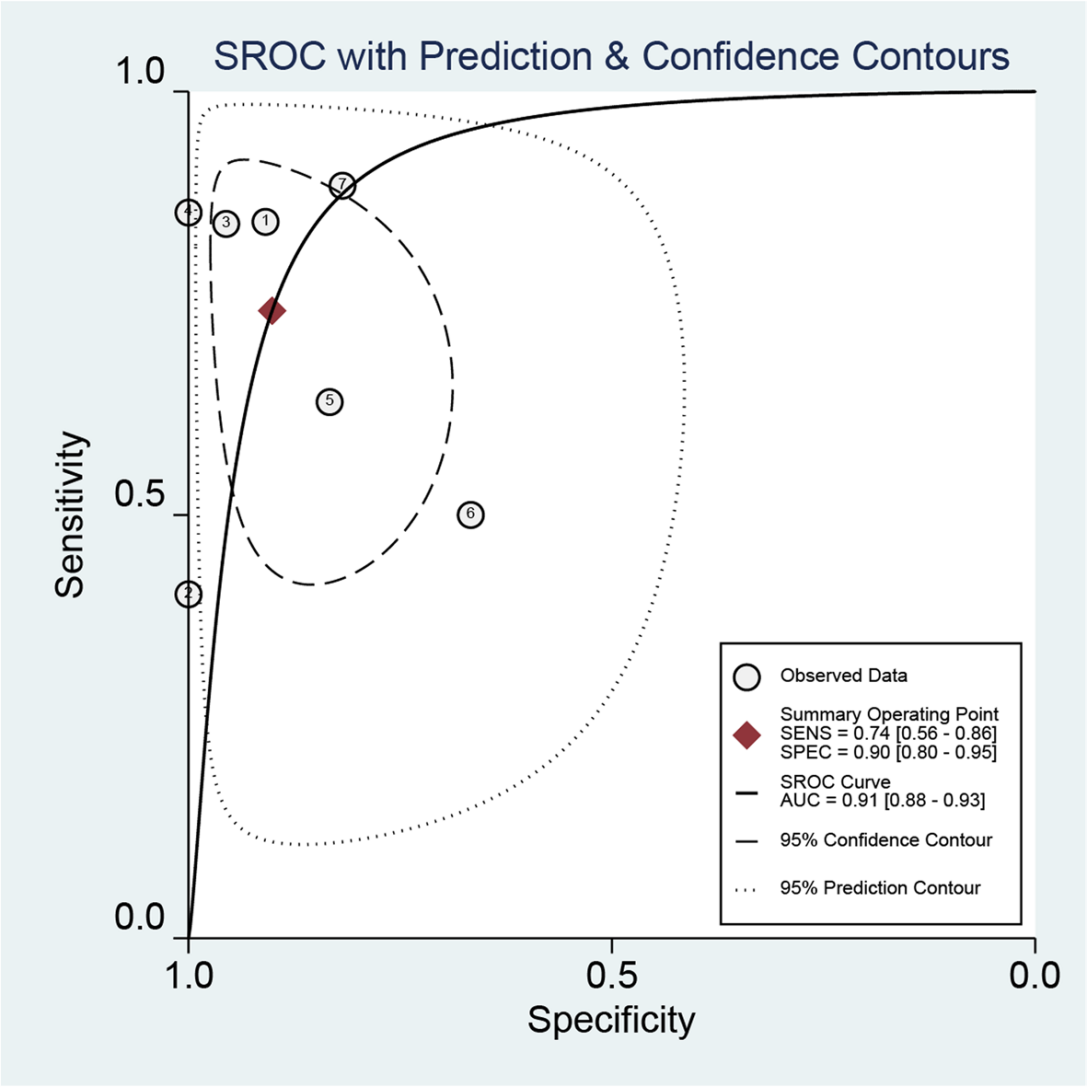
Summary receiver operating characteristic (SROC) curve of the diagnostic accuracy of EpCAM. The SROC curve showed comphensive consideration of diagnostic threshold based on sensitivity and specificity. Every hateched circle mean every single study’s predictive region of summary operating characteristic 95% CI. Area under curve (AUC) was an diagnostic accuracy index of which value between 0.8 and 1 represented relatively high for clinical consideration

An overview of the sensitivity and specificity of EpCAM in the detection of recurrence or metastases among TC patients is displayed in Fig. 3. Seven studies investigated the diagnostic accuracy of EpCAM in 307 participants. There was heterogeneity shown in meta-analysis of EpCAM, as revealed by the results of sensitivity (*p* = 0.0005, *I*^2^ = 74.9%) and specificity (*p* = 0.02, *I*^2^ = 60.1%), respectively. Thus, random effects models were adopted for analysis and the DOR was 26.75 (95%CI: 9.11–78.53) (Fig. 4). The pooled positive likelihood ratio was 6.19 (95% CI: 3.05–12.57), and the pooled negative likelihood ratio was 0.30 (95% CI: 0.15–0.57). Of note, the area under curve (AUC) of the SROC plot was employed to assess the overall accuracy of a diagnostic test. The SROC curve of EpCAM produced an AUC of 0.91 (Fig. 5), which showed that the overall performance of EpCAM was suitable.

**Fig. 6:**
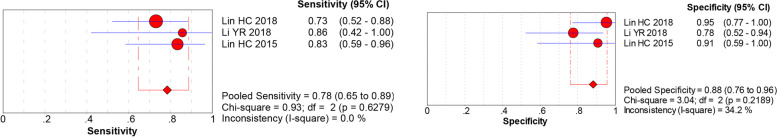
Forest plot of reported sensitivity and specificity of thyroide-stimulateing hormone receptor (TSHR). Left forest plot was for sentivity of TSHR and right forest plot was for specificity of TSHR in studies

Three studies investigated the diagnostic accuracy of TSHR in 102 participants. This meta-analysis of TSHR for detecting the recurrence of TC generated a pooled sensitivity of 0.88 (95%CI: 0.76–0.96) and pooled specificity of 0.78 (95%CI: 0.65–0.89) (Fig. 6). The AUC of SROC curve is 0.91. The pooled DOR for the TSHR was 40.01 (95%CI: 10.49-152.63) (Fig. 4), thus showing the high discriminative power.

The Youden index, indicating the diagnostic test accuracy, calculated as (sensitivity + specificity − 1). A diagnostic test is supposed to be suitable if its Youden index is above 0.5. The Youden index of the TSHR is 0.66, which was compared with EpCAM (0.6). Based on the comparative outcomes of the AUC and Youden index for detecting the recurrence or metastases, EpCAM and TSHR were both excellent choices.

### Evaluation of threshold effect and heterogeneity

To evaluate the possible reasons of high heterogeneity, the threshold effect was firstly considered. For biomarker of EpCAM, no evident threshold effect was presented in this meta-analysis, supported by the value of proportion of heterogeneity likely due to threshold effect (0.25).

Given study design, socio-demographic characteristics and geographical location might also represent sources of the heterogeneity, we conducted a meta-regression analysis with geographical location, quality of study, study design and detection methods include in Table 2. No significant heterogeneity was suggested with respect to region (coefficient=-1.118, *p* = 0.2360), quality of study (coefficient = 0.036, *p* = 0.7824), study design (coefficient = 0.201, *p* = 0.8996) and detection methods (coefficient = -1.862, *p* = 0.5867).Thus, other factors might contribute to the observed high heterogeneity.

**Table 2:** Results of meta-regression on EpCAM

Variable	Coefficient	Standard Error	*P* value	RDOR
Region	-1.118	0.4346	0.2360	0.33
Detection method	-1.862	2.4535	0.5867	0.16
Quality of study	0.036	0.7824	0.9708	1.04
Study design	0.201	1.2607	0.8996	1.22

*P* < 0.05 was considered as to be statistically significant.

### Publication bias

**Fig. 7:**
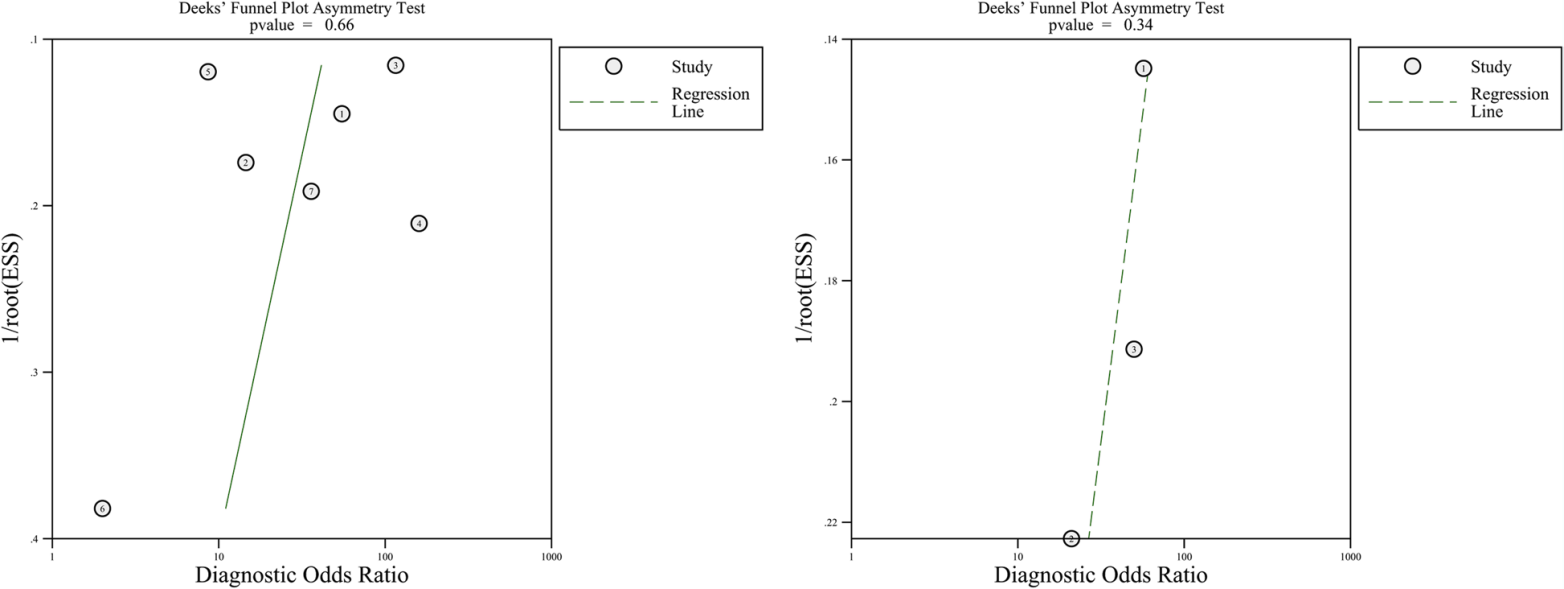
Deek’s plots for the assessment of publication bias. Left Deek’s plot was for publication bias of Epcam and right Deek’s plot was for that of TSHR in studies. ESS represented effective sample size of every study. Symmetrical funnels and*p* value of both EpCAM and TSHR were above 0.05, suggesting that there was no publication bias

Potential publication bias was assessed by Deeks’ funnel plots. Note that, *p* < 0.05 indicated the existence of publication bias. There was no evidence of publication bias for the pooled analysis of EpCAM (*p* = 0.66) and TSHR (*p* = 0.34). The funnel plots of publication bias on EpCAM and TSHR are shown in Fig. 7.

## Discussion

Thyroid cancer (TC) is a malignant tumor originating from the epithelium of the thyroid gland. Although the overwhelming majority of the TC patients had a satisfied prognosis, it is estimated that few patients will develop into metastases and eventually succumb to their disease even after adopting advanced and comprehensive therapeutic approaches [22]. CTCs play an important role in the diagnosis, treatment, and prognosis of malignant tumors and have unique advantages compared with traditional diagnostic methods. In recent years, with the rise of molecular biology, numerous molecular biomarkers and single nucleotide polymorphism genotyping have shed new light on discovery of diseases and provide valuable information for clinical decision making [23, 24]. Although CTCs detection in TC patients is essential in order to observe the treatment effect, establish prognosis and prevent unnecessary surgery, the prognostic value of CTCs on TC remains unclear and does not attract enough attention.

Since CTCs were first identified in peripheral blood of cancer patients, the clinical value of CTCs had become a debated topic throughout the medical community. The poor prognosis of CTC-positive patients has been confirmed in some solid tumors [25–27]. A large cohort study focused on breast cancer did not prove the prognostic values of CTCs with an immunocytochemistry method [28]. However, a pooled analysis indicated that esophageal cancer patients with positive CTCs were associated with high recurrence and poor response of radiotherapy and chemotherapy [29]. In our meta-analysis, the AUC both above 0.90 showed that CTCs may be a promising biomarker to discriminate recurrent TC patients from remission individuals, with high sensitivity and specificity, and increased counts of EpCAM+-CTCs or TSHR+-CTCs could be highly suggestive of recurrent disease or disease in progression.

Although we set restrict inclusion criteria to reduce the heterogeneity, no significant heterogeneity was found in the pooled analysis of TSHR. Nevertheless, for EpCAM, there was still a certain extent of heterogeneity in our meta-analysis. Heterogeneity may derive from differences in the publication year, country and quality of publication, along with differences in sample capacity. In addition, differences in the detection method also generated non-ignorable heterogeneity. Thus, we conducted a meta-regression to seek the source of heterogeneity, no reason was found. The real cause of heterogeneity could result from other factors that we failed to extract from the studies.

Currently, substantial controversy exists regarding the optimal diagnostic criteria of TC recurrence or DMs. In clinical practice, the post-operative evaluation of TC with recurrence mainly depends on Tg, TgAb assay and medical imaging. However, Tg assay always operated in TSH depressed condition and ^131^I WBS increased the adverse effects by radiation to the human body. Thus, molecular markers are urgently necessary for this purpose. Different subtypes of CTCs are present in the blood stream. In addition to the common epithelial cell marker EpCAM, CTCs expressing TSHR were identified in the peripheral blood of patients with TC [30]. TSHR, a G protein-coupled receptor, has been reported mainly expressed in thyroid follicular cells type. Although TSHR is also presented in the cells of non-thyroid origin, such as fat, bone, and muscle with little expression, this kind of thyroid-related proteins still helps us to define the thyroid origin of CTCs because TSHR is a general marker for the cells with thyroid origin and has a minimal impact on their use as prognostic markers for TC patients [31].

This meta-analysis has several limitations that need to be considered. First and foremost, as a novel biomarker, CTCs for the diagnosis of TC recurrence or DMs has been conducted in limited studies. Therefore, no large number of patients can be included in this meta-analysis. Furthermore, we supposed CTCs combined with Tg and medical imaging should have better performance than anyone alone. Unfortunately, these studies were too few for further analysis. Second, there was considerable heterogeneity in our study. Although we rule out the causes from geographic location, sample capacity, and detection method, the resource of heterogeneity was unidentified due to the limited variables available. However, we addressed the heterogeneity among studies by using a random effects model to obtain more conservative estimates. Third, eligible studies were restricted to those written in English according to language criteria, which may cause language bias and generate an overestimation of effect sizes. Despite these limitations exist, our meta-analysis was the first study to assess the prognostic significance of CTCs in TC patients and indicate that CTCs could also supplement other clinical factors or markers such as Tg in clinical surveillance of disease status. However, future large-scale clinical studies are inevitable to validate this finding.

## Conclusion

 CTC was a reliable marker for the diagnosis of TC patients with recurrence and DMs.

## Data Availability

All data generated or analyzed during this study are included in this article.

## Abbreviations

APC: annual percent change
AUC: area under curve
CECs: circulating epithelial cells
CI: confidence interval
CTCs: circulating tumor cell
DMs: distant metastasis
DOR: diagnostic odds ratio
EpCAM: epithelial cell adhesion molecule
FN: false negatives
FP: false positives
MRI: magnetic resonance imaging
QUADAS-2: Quality Assessment of Diagnostic Accuracy Studies-2
SROC: summary receiver operating characteristic
TC: thyroid carcinoma
Tg: thyroglobulin
TgAb: Tg antibody
TN: true negatives
TP: true positives
TSHR: thyroid stimulating hormone receptor
^131^I-WBS: ^131^I-whole body scintigraphy
